# Transcriptomic Impact of IMA-08401, a Novel AHR Agonist Resembling Laquinimod, on Rat Liver

**DOI:** 10.3390/ijms20061370

**Published:** 2019-03-19

**Authors:** Stephenie D. Prokopec, Raimo Pohjanvirta, Selma Mahiout, Lars Pettersson, Paul C. Boutros

**Affiliations:** 1 Ontario Institute for Cancer Research, Toronto, ON M5G 0A3, Canada; Stephenie.Prokopec@oicr.on.ca; 2 Laboratory of Toxicology, National Institute for Health and Welfare, FI-70210 Kuopio, Finland; raimo.pohjanvirta@helsinki.fi; 3 Department of Food Hygiene and Environmental Health, University of Helsinki, FI-00790 Helsinki, Finland; selma.mahiout@ttl.fi; 4 Immunahr AB, SE-22480 Lund, Sweden; larspettersson59@live.se; 5 Department of Pharmacology & Toxicology, University of Toronto, Toronto, ON M5S 1A8, Canada; 6 Department of Medical Biophysics, University of Toronto, Toronto, ON M5G 1L7, Canada; 7 Department of Human Genetics, University of California, Los Angeles, CA 90095, USA; 8 Department of Urology, University of California, Los Angeles, CA 90095, USA; 9 Institute for Precision Health, University of California, Los Angeles, CA 90095, USA; 10 Jonsson Comprehensive Cancer Center, University of California, Los Angeles, CA 90095, USA

**Keywords:** AHR, laquinimod, TCDD, immunomodulator

## Abstract

IMA-08401 (C2) is a novel aryl hydrocarbon receptor (AHR) agonist and selective AHR modulator (SAHRM) that is structurally similar to laquinimod (LAQ). Both compounds are converted to the AHR-active metabolite DELAQ (IMA-06201) in vivo. SAHRMs have been proposed as therapeutic options for various autoimmune disorders. Clinical trials on LAQ have not reported any significant toxic outcomes and C2 has shown low toxicity in rats; however, their functional resemblance to the highly toxic AHR agonist 2,3,7,8-tetrachlorodibenzo-*p*-dioxin (TCDD) raises questions. Here, we characterize the hepatic transcriptomic changes induced by acute (single-dose) and subacute exposure (repeated dosing for 5 days followed by a 5-day recovery period) to C2 in Sprague-Dawley rats. Exposure to C2 leads to activation of the AHR, as shown by altered transcription of *Cyp1a1*. We identify a heightened response early after exposure that drops off by day 10. Acute exposure to C2 leads to changes to transcription of genes involved in antiviral and antibacterial responses, which highlights the immunomodulator effects of this AHR agonist. Subacute exposure causes an oxidative stress response in the liver, the consequences of which require further study on target tissues such as the CNS and immune system, both of which may be compromised in this patient population.

## 1. Introduction

Laquinimod (LAQ) is a derivative of linomide (also known as roquinimex). Linomide has immunomodulatory, antiangiogenic, and antineoplastic activity, and was once proposed as a therapy for cancer and autoimmune diseases because it can both enhance and curb immune responses [1,2,3]. Early clinical trials, however, demonstrated considerable toxic effects and were halted [4]. Compared with linomide, LAQ demonstrates reduced toxicity, but has proven effective in mouse models of autoimmune disorders, functioning as an immunoregulatory drug without general immunosuppressive properties [5]. Since these models share considerable clinical and immunological characteristics with human autoimmune disorders, LAQ has been proposed for treatment of multiple sclerosis (MS) [6,7], Crohn’s disease [8], and Huntington’s disease [9]. Early studies in MS demonstrated a favorable safety profile and effective treatment, with patients demonstrating reduced lesion development, brain atrophy, and disease activity in the relapsing–remitting disease type [7,10]. Multiple phase III clinical trials have been completed; however, final results are as yet unavailable [11,12,13].

The immunomodulatory effect of LAQ is believed to result from activation of the aryl hydrocarbon receptor (AHR) [9,14]. Early experiments demonstrated that typical ‘AHR-core’ genes (genes consistently upregulated by AHR activation, including multiple xenobiotic metabolism enzymes such as *Cyp1a1* and *Cyp2a1* [15,16]) were induced in experimental autoimmune encephalomyelitis (EAE) mice treated with LAQ [9]. AHR-null EAE mice showed no benefit from LAQ therapy, despite similar CNS immune infiltration profiles [9,14]. Furthermore, the therapeutic effects of LAQ could be recovered in these mice through bone marrow transplant from wild type AHR-wild type EAE mice [9], indicating that LAQ is a potent agonist of immune cell AHR, and this activation is required to produce therapeutic effects.

The AHR is a well-described transcription factor, with a key physiological role in xenobiotic metabolism, regulation of cell growth and differentiation and immune modulation. When inactive, it resides in the cytoplasm, bound to chaperone proteins [17]. Ligand-binding induces a conformational change within the protein, causing it to translocate to the nucleus and dissociate from its chaperone proteins. Once in the nucleus, the AHR binds with the AhR-nuclear translocator (ARNT) [17]. This AHR-ARNT dimer then binds specific recognition motifs on DNA, called AHR response elements (AHREs) and can regulate the transcription of target genes [18]. In particular, this complex will consistently induce (or repress) expression of a collection of genes, deemed the ‘AHR-core’ battery of genes [15,16,19,20,21,22]. In addition to this canonical signaling pathway, the AHR can also influence gene expression by alternative pathways including epigenetic mechanisms (e.g., DNA methylation, histone modifications, miRNA, and noncoding long-RNA induction as reviewed in [23,24,25]), functioning as a nuclear E3 ubiquitin ligase, and cross-talk with other transcription factors [26,27,28,29].

The function of the AHR has been substantially characterized through studies of 2,3,7,8-tetrachlordibenzo-*p*-dioxin (TCDD); the most potent dioxin congener known. Exposure to TCDD produces a wide variety of toxic outcomes. In model organisms, these include chloracne, hepatotoxicity, immune suppression, thymic atrophy, wasting syndrome, and cancer [30]. In humans, the most consistent sign of high TCDD exposure is chloracne; however, numerous studies have linked exposure to cancer incidence and mortality [31,32,33,34] and other morbidities, such as diabetes [35,36,37]. These outcomes can be directly linked to TCDD-activation of AHR—numerous studies have demonstrated differences in TCDD-induced toxicity between and within species resulting from genetic differences affecting AHR structure. AHR-null mice are refractory to all toxicities [38,39], while DBA/2 mice and Han/Wistar (*Kuopio*; H/W) rats show considerable resistance relative to their wild type counterparts, due to polymorphisms within the *Ahr* gene [40].

LAQ is metabolized partly by N-dealkylation to give the potent AHR agonist DELAQ (deethylated LAQ; IMA-06201) in minute amounts [41]. The prodrug IMA-08401 (henceforth referred to as C2) is a diacetyl prodrug of DELAQ, representing a novel selective AHR modulator designed to effectively hydrolyze to DELAQ in vivo (to a far greater extent than LAQ) and being a potent agonist of the AHR [42]. Rats exposed to C2 demonstrated increased mRNA abundance of multiple ‘AHR-core’ genes across multiple tissue types, similar to that observed following exposure to TCDD [42]. Interestingly, exposed animals did not exhibit most of the major signs of toxicity typically observed with TCDD exposure, despite repeated daily administration of high C2 doses (highest achievable given the solubility characteristics of the compound). Perhaps most strikingly, these rats did not experience the dramatic body weight loss associated with TCDD-induced wasting syndrome [42,43].

To elucidate the reasons for these differences, we characterized the hepatic transcriptomic profile of C2-exposed Sprague-Dawley rats, focusing on the liver as a primary site of TCDD-mediated toxicities [30,40]. The transcriptomic profiles from acute and subacute exposures have been compared, and results contrasted with those from similar studies of TCDD exposure. This study will provide valuable insight into the mechanism and safety of C2 and provide a basis of comparison for other proposed therapeutic AHR agonists.

## 2. Results

### 2.1. Experimental Design

**Figure 1:**
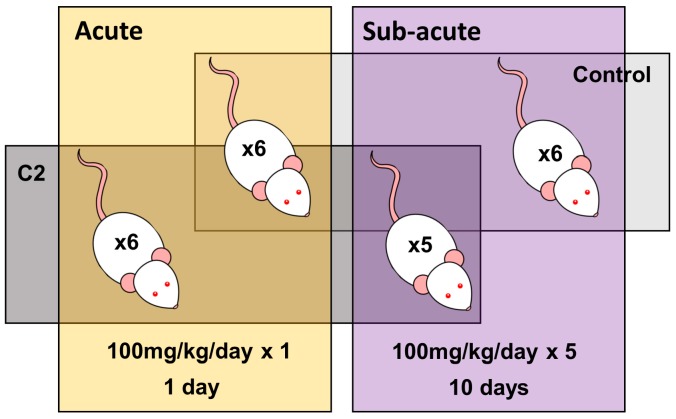
Experimental design. Twenty-three adult male Sprague-Dawley rats were divided into 2 experimental groups. The acute treatment group was treated with a single dose of either 100 mg/kg C2 dissolved in vehicle, or vehicle alone, with liver samples collected 1 day after treatment. The subacute treatment group was treated with repeated doses (daily for 5 consecutive days) of either C2 (100 mg/kg) or vehicle, with tissue samples collected 10 days after the initial treatment.

In order to evaluate the toxicity of acute and subacute exposure to C2—a prodrug that is readily hydrolyzed to form the AHR-active LAQ metabolite DELAQ in vivo—two separate experiments were carried out in adult male Sprague-Dawley rats: an acute study and a subacute one (Figure 1, Table S1). In the acute study, rats were treated once with either 100 mg/kg C2 dissolved in vehicle (PEG-400) or vehicle alone, and euthanized 1 day after exposure. In the subacute study, rats received daily either 100 mg/kg C2 dissolved in PEG-400 or vehicle alone for five consecutive days, and were euthanized after a recovery period of five days on experimental day 10. As liver was previously shown to be susceptible to C2-induced *Cyp1a1* mRNA abundance [42], and is one of the primary sites of TCDD-mediated toxicities [30,40], hepatic tissue was excised for transcriptomic profiling. For comparison of the transcriptomic impacts of C2 and TCDD, data previously generated from hepatic tissue from TCDD-sensitive Long–Evans (*Turku/AB*; L–E) and TCDD-resistant H/W rats, following a single exposure to 100 µg/kg TCDD and collected at 19 h or 10 days afterwards were used [44,45].

### 2.2. Transcriptomic Profiles of Acute and Subacute Treatments Differ Considerably

The mRNA abundances of 21,219 total transcripts were measured using Affymetrix microarray technology. The mRNA abundance profiles of the most variable genes, defined as variance > 1 across the combined cohort, differed considerably between animals with different dosing regimens (acute vs. subacute) rather than between treatment type (C2 vs. control; Figure S1). These differences might be attributable to differences in recovery time after the end of the exposure period, rapid metabolism and elimination of C2, prolonged stress from repeated dosing, housing in groups (subacute) vs. individually (acute), or secondary events downstream of AHR activation.

**Figure 2:**
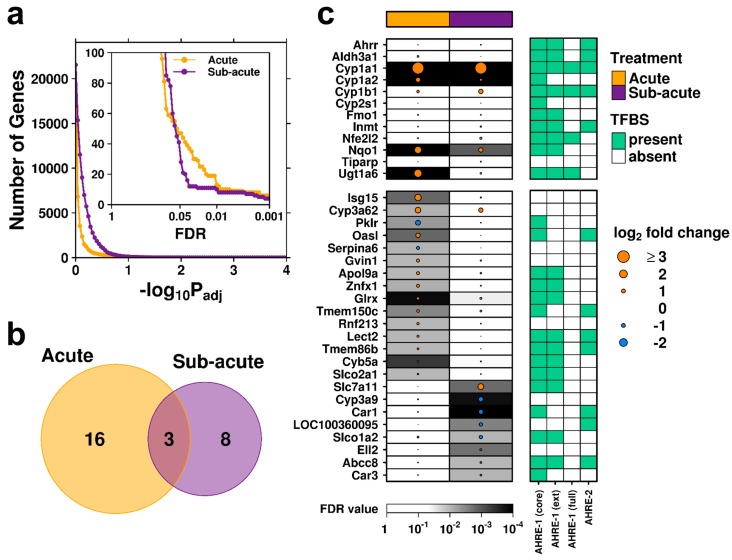
Transcriptomic profiles. (**a**) Following linear modeling, sensitivity to various FDR-adjusted *p*-value cut-offs was examined; yellow indicates genes determined to be significant in liver tissue from rats in the acute treatment group, while purple represents animals in the subacute treatment group. A FDR-threshold of 0.01 was selected, and genes with differentially abundant mRNA compared between treatments. (**b**) Venn diagram demonstrating the overlap of differentially expressed genes. (**c**) The magnitude and direction (log_2_-fold change) for genes with significantly differentially abundant mRNA, along with the standard ‘AHR-core’ gene battery are shown; dot size indicates magnitude of change, color indicates direction of change and background shading indicates statistical significance (FDR). Covariates (right) indicate presence (black) or absence (white) of described transcription factor binding sites.

With these differences in mind, linear modeling was performed with contrasts to identify differentially abundant transcripts in C2-exposed liver relative to control specimens for the acute and subacute exposure groups separately. Rats in the acute exposure group demonstrated a more pronounced transcriptomic perturbation relative to controls (Figure 2a, yellow curve, at statistically relevant thresholds) than the subacute exposure group (purple curve), plausibly reflecting the 5-day recovery period in the subacute study. A statistical threshold of FDR < 0.01 was used to define transcripts with significantly differentially abundant RNA; this identified 19 genes in the acute group and 11 in the subacute group (Figure 2b). Three genes (*Cyp1a1*, *Cyp1a2*, and *Nqo1*) were found to have altered mRNA abundance in the same direction in both groups. These represent xenobiotic response genes and members of the ‘AHR-core’ battery [15,16,19,20,21,22], suggesting that C2 exposure leads to hepatic AHR activation (Figure 2c, top panel).

### 2.3. Rapid Recovery Observed Following Subacute Exposure to C2

**Figure 3:**
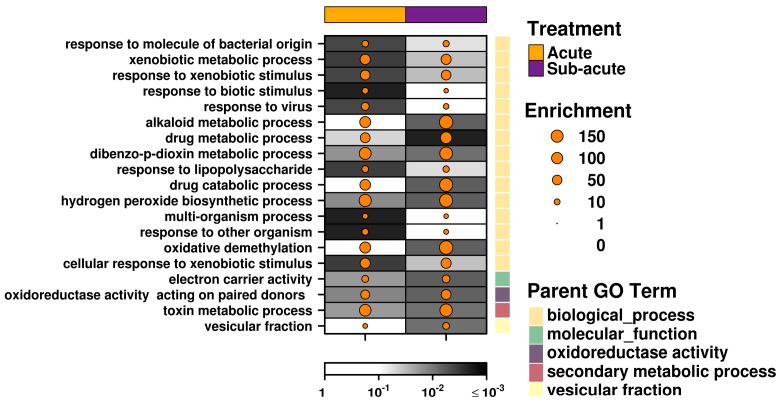
Pathway analysis. Genes which demonstrated significantly altered mRNA abundance (FDR < 0.05 and |log_2_ fold change| > 0.5) following exposure to C2 were evaluated for pathway enrichment analysis. Pathways which showed significant enrichment (FDR < 0.01 and enrichment > 5 genes) in either the acute or subacute treatment groups are shown.

Apart from the above mentioned ‘AHR-core’ genes, acute and subacute exposure to C2 produced strikingly different changes to the transcriptome. Sixteen genes showed altered mRNA abundance following acute exposure, but showed no significant changes after subacute exposure. A subset of genes, including *Cyp3a62*, *Glrx*, and *Tmem150c*, showed a similar trend, but this was not typical. Conversely, eight genes showed significant changes in the subacute group that were not apparent following acute exposure (Figure 2c, bottom panel). Importantly, the majority of these genes were found to contain AHREs within 3 kb of the transcription start site. This fact and the dissimilar mRNA abundance profiles after two dosing regimens with different durations for exposure and recovery are consistent with a model of swift AHR activation by the canonical pathway, followed by rapid metabolism and removal of the available ligand and a gradual return to an inactive state. A pathway analysis using less stringent thresholds to expand the available gene lists provides further support: both treatment groups showed transcriptomic responses enriched for genes in pathways associated with xenobiotic metabolism, with secondary processes surrounding a response to oxidative stress more apparent in the subacute treatment group (Figure 3, Table S2).

### 2.4. Acute Exposure to C2 May Stimulate an Immune Response

Intriguingly, a number of immune-related genes demonstrated significant changes in mRNA abundance following acute exposure to C2 that were not found following subacute exposure (Figure 2c). In particular, interferon-stimulated gene 15 (*Isg15*) had a statistically significant 3.1-fold increase in mRNA abundance following exposure to C2 (FDR = 0.0015), and has been shown to be a key mediator of the antiviral and antimycobacterial response [46,47,48,49]. Similarly, 2′-5′-oligoadenylate synthetase-like (*Oasl*) mRNA showed a significant 2.2-fold induction (FDR = 0.0015), and is also involved in the antiviral response mechanism [50,51]. *Gvin1* (GTPase, very large interferon inducible 1) also demonstrated increased mRNA abundance in the acute-C2 treated rats (FDR = 0.0097); however, this may be a consequence of increased interferon levels [52]. To further support this, pathway analysis identified a significant enrichment for genes involved in the response to virus pathway (Figure 3). This pathway includes viral response genes *Mx1* (2-fold increase, FDR = 0.043), *Oas1b* (1.95-fold increase, FDR = 0.032), and *Rsad2* (1.74-fold increase, FDR = 0.020), all of which show near significant induction.

In addition to an antiviral response, additional immune-related pathways, including response to molecule of bacterial origin, response to biotic stimulus and response to other organism, were all significantly enriched in the acute-C2 exposed group (Figure 3). These pathways involve many of the above described genes, as well as pyruvate kinase L/R (*Pklr*), which showed significantly reduced mRNA abundance in C2-treated rat liver (Figure 2c). Deficiency of *Pklr* has been shown to have a protective role against malaria [53], but is more known for its involvement in glycolysis. Serpin family A member 6 (*Serpina6*; also known as corticosteroid-binding globulin), a biomarker of inflammatory processes in rats [54], showed significantly reduced mRNA abundance (Figure 2c). Finally, glutaredoxin (*Glrx*) is part of the antioxidant defense system and demonstrated significantly increased mRNA abundance (Figure 2c).

### 2.5. Subacute Exposure to C2 Leads to Oxidative Stress

Relative to the acute-C2 exposure group, the rats in the subacute group demonstrated changes in RNA abundance of genes with potentially toxic outcomes. Eight genes demonstrated significant changes in RNA abundance in liver from subacute C2 exposure alone (Figure 2c, bottom panel). Solute carrier family 7 member 11 (*Slc7a11*) exhibited the largest effect size—2.9-fold (FDR = 0.0016); this is a cysteine/glutamate antiporter typically not expressed in rat liver, but shown to be induced under conditions of oxidative stress, to increase glutathione levels [55]. Interestingly, this transporter system has been shown to enhance viral entry into the cell [56], and is increased in the CNS of patients with MS [57]. *Cyp3a9* demonstrated significantly reduced mRNA abundance, but the absence of AHREs in the promoter of this gene suggests it is an indirect effect or mediated by an alternative route of AHR signaling. Carbonic anhydrase 1 and 3 (*Car1* and *Car3*, Table 1) showed significantly reduced mRNA abundance (44% and 72% of control, respectively); of these, *Car3* is typically expressed in liver, contains four occurrences of the core AHRE motif in its promoter region and has been shown to be reduced during hepatocarcinogenesis [58]. Taken together, subacute exposure to C2 led to transcriptional changes associated with a heightened response to oxidative stress (Figure 3). This suggests that even short-term exposure to C2 may be harmful.

### 2.6. Exposure to C2 Leads to a Drastically Different Transcriptomic Profile From the Prototypical AHR Agonist TCDD

**Figure 4:**
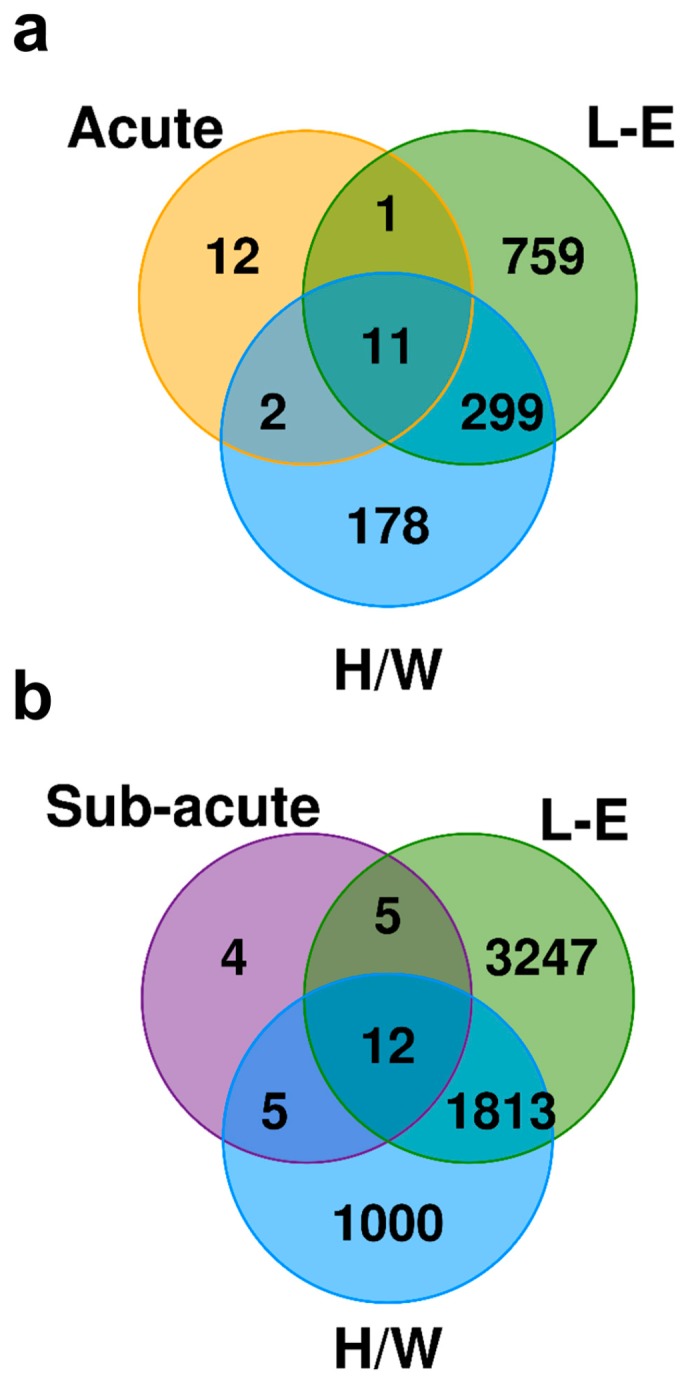
Transcriptomic impact of C2 differs from that of TCDD. Venn diagrams display the number of genes which had significantly different mRNA abundance following (**a**) acute exposure to C2 (Sprague-Dawley rats) and TCDD (H/W or L-E rats) or (**b**) subacute exposure to C2 (repeated dosing) and TCDD (single dose). Only genes with mRNA abundance measured in both studies were considered (*n* = 9534).

In comparison, TCDD-induced activation of the AHR leads to drastically different changes in mRNA abundance in rat liver under similar experimental conditions [45,59]. As the study of TCDD effects was performed using a different setting, and a considerable number of genes were not available on both platforms (only a 9534 gene overlap), a less stringent significance threshold of FDR < 0.05 was used to consider overlap across studies. Shortly after a single exposure to either C2 or TCDD, 11 genes demonstrated changes to RNA abundance in the same direction with similar magnitudes (Figure 4a); these include a number of ‘AHR-core’ genes (*Cyp1a1*, *Cyp1a2*, *Nqo1*, and *Cyb5a* [60]), as well as *Glrx* and *RGD1310209* (a gene similar to *EIG121* that is involved in regulation of autophagy). Similarly, 10 days after initial exposure to either C2 or TCDD, 12 genes showed changes to RNA abundance of similar size and direction, again including *Cyp1a1*, *Cyp1a2*, *Nqo1*, *Glrx*, and *RGD1310209* (Figure 4b). Perhaps more interesting are those genes uniquely altered by C2 exposure. Acute exposure to C2, but not TCDD, led to transcriptional changes of genes involved in viral and bacterial responses, as described above. Prolonged exposure to C2 produced changes to the mRNA abundance of *Cebpz*, *Lox*, *Pdk3*, and *Strn* that were not observed following exposure to TCDD. Alternatively, subacute exposure to C2 produced significantly reduced mRNA abundance of both carbonic anhydrase 1 and 3 (*Car1* and *Car3*): *Car1* was repressed in the TCDD-resistant H/W rat liver as early as 19 h and continuing to at least 10 days after exposure (down to 21% of control), whereas *Car3* was repressed in the liver of TCDD-sensitive L-E rat under identical conditions even more severely (3% of control by 4 days; Table 1). Hence, C2 and TCDD produce both common and distinct transcriptional changes, the consequences of which require further study.

## 3. Discussion

C2 represents a novel selective AHR modulator with considerable structural similarities to the AHR agonist LAQ that is currently under study as a therapeutic option for autoimmune disorders. Compared with LAQ, C2 generates much greater amounts of the AHR-active metabolite, DELAQ. To date, no overt toxic outcomes have been reported from clinical trials on LAQ. However, as these compounds act through ligand activation of the AHR, concerns regarding their safety exist. These concerns revolve around the similar structure and mechanism of action of these compounds to those of the most potent AHR-ligands—dioxins, and in particular, TCDD [42]. Our recent in vitro studies with the active derivative of C2 proved it to have virtually equal potency in inducing CYP1A1 activity to TCDD in a rat hepatoma cell line and exhibit similar modelled binding properties to the ligand binding region of the AHR [61]. However, despite these similarities, our in vivo studies in rats demonstrated that C2 elicits only a fraction of the biological impacts of TCDD, with one of them being *Cyp1a1* gene induction. The most notable difference concerned grave toxicities which were lacking in C2-treated rats [42]. The subacutely exposed liver samples analyzed here originate from that study.

To gain further insight into C2′s effects in vivo, the transcriptomic profiles of Sprague-Dawley rat liver exposed to C2 or vehicle were examined and further compared with transcriptional changes produced by TCDD. In addition to single administration, we also examined effects of subacute exposure with daily treatments due to the fact that this potentially therapeutic AHR agonist is rapidly metabolized and excreted (elimination half-life in rats 1.7 h after C2 per oral dosing at 0.5 mg/kg; unpublished data). In contrast, TCDD is recalcitrant to biotransformation and has a half-life of three weeks in both TCDD-resistant and TCDD-sensitive rat strains [62], which enables long-term effects by a single exposure. In fact, we identified a drastically different transcriptional response pattern following exposure to C2 and TCDD in rat liver at two time points (Figure 4), despite both compounds producing changes in mRNA levels of key ‘AHR-core’ genes. This is not without precedence for potent AHR agonists. For example, in mice at toxic equivalence factor-adjusted equipotent hepatic levels, 2,3,7,8-TCDF and PCB-126 altered the expression levels of only subsets of the genes affected by TCDD [63,64]. The differences observed between C2- and TCDD-mediated transcriptional changes among the ‘AHR-core’ genes may be the result of fewer secondary effects, such as activation of the Nrf2 transcription factor [20], due to the reduced half-life of C2. Importantly, the degree of overlap was similar between C2 and TCDD for both TCDD-sensitive and TCDD-resistant strains of rat, suggesting the reduced response is not due to resistance. When considering only those genes evaluated in both studies, the number of genes whose expression was significantly altered was also conspicuously low in rats treated with C2 as compared with rats administered TCDD. This is in keeping with the low hepatic toxicity of C2 established by histological analysis in our previous study [42].

Of particular interest, we identified a distinct set of genes altered by acute exposure to C2 that were not detected following subacute exposure, or following exposure to TCDD. Intriguingly, these are genes which have roles in modulation of the immune system, in particular, the response to viral and bacterial infection and inflammation. The altered expression patterns of these genes are unlikely to have stemmed from the microbial status of the rats because the animal facilities were strictly controlled to be free of specific pathogens. On the other hand, at least a contributing factor to the outcome is probably the longer recovery time after subacute C2 exposure because of the fairly swift elimination of the compound. As the changes recorded in expression patterns of these genes theoretically imply an enhanced immune response, the outcome may appear surprising, because the primary therapeutic goal of LAQ and C2, with regards to treatment of autoimmune disorders, is to suppress the immune response. However, here we analyzed transcriptomic responses in the liver, not in immune cells. It should also be noted that classification of an agent regarding its effects on immune defense is not always straightforward. Although TCDD is a notorious immune suppressant, it protects mice against *Streptococcus pneumoniae*-induced mortality and reduces pulmonary bacterial burden; it also mitigates the severity of leishmania disease in mice, independent of adaptive immunity [65,66]. Furthermore, in the immune system AHR has been demonstrated to be capable of directing naïve CD4+ T cell differentiation into either proinflammatory Th17 cells or into anti-inflammatory Treg cells, with the dose (or dosing) and duration of AHR activation by high-affinity ligands being the primary drivers of the direction [67]. Thus, the very rapidly biodegraded physiological AHR agonist 6-formylindolo[3,2-b]carbazole (FICZ) can both mitigate and aggravate EAE in mice depending on its dose [68,69,70]. The influence on EAE of C2 itself, with a 1.7 h per oral half-life of the AHR active metabolite, has, in fact, also been tested. Sc. administration of 1 mg/kg C2 on days 0, 3, 6, and 9 after EAE induction in rats was found to efficiently prevent EAE development (unpublished data). Therefore, further studies should be performed to assess these effects in a more relevant tissue, such as cells of the immune system, rather than liver.

On the other hand, pathways enriched more significantly or in a greater magnitude by the subacute than acute exposure included oxidoreductase activity, hydrogen peroxide biosynthetic process, oxidative demethylation, and electron carrier activity, collectively pointing to a response to oxidative stress. This is not unexpected, because oxidative stress is a common and, largely, tissue-independent consequence of TCDD exposure in rodents [71], and production of reactive oxygen species is synergistically augmented in the simultaneous presence of FICZ and ultraviolet A irradiation [72,73]. Interestingly, the gene showing the greatest increase in its expression level (*Slc7a11*) was also induced by LAQ in mouse splenocytes 6 days after treatment [9]. Whether the degree of oxidative stress in C2-treated animals is substantial enough to result in functional or morphological tissue damage over time requires further study, but—as mentioned above—at least histological examination of the livers of the rats used in the subacute study did not reveal any grave lesions [42], as would be observed in TCDD-exposed rat liver over a shorter time frame [74].

In the present study we report transcriptomic profiling of rat liver following acute and subacute exposure to a rapidly metabolized AHR agonist termed C2. Exposure to C2 led to activation of the AHR, as shown by changes in mRNA abundance of ‘AHR-core’ genes, in particular, *Cyp1a1*, with a heightened response shortly after exposure that reduced over time. Subacutely, C2 further elicited a pattern of changes in gene expression typical of oxidative stress in the liver, and appeared to provoke a significant immune response, with a focus on antiviral and antibacterial responses. However, in spite of the high doses of C2 applied, the number of genes affected in the liver remained overall very low. Further studies must be performed to understand the consequence of C2 exposure on other tissues such as the CNS and immune system, and to further assess potential toxic outcomes of chronic exposure.

## 4. Materials and Methods

### 4.1. Animal Handling and Sample Preparation

In vivo studies were authorized by the National Animal Experiment Board in Finland (Eläinkoelautakunta, ELLA; project license codes ESAVI/6882/04.10.03/2012, ESAVI/217/04.10.07/2016 and ESAVI/3436/04.10.07/2017). All procedures were conducted in a humane manner and in accordance with the Directive 2010/63/EU of the European Parliament and the Council. All animal handling and reporting comply with ARRIVE guidelines [75].

A total of 24 young adult (~9 weeks of age) male Sprague-Dawley rats were purchased from Harlan (Netherlands) and acclimatized to their new surroundings for at least a week before the onset of the studies. In the course of the studies, they were housed in individually ventilated plastic cages (Sealsafe IVC Blue Line or Green Line IVC Sealsafe PLUS Rat, Techniplast, West Chester PA, USA), and maintained on a 12-h light/dark cycle (06:00−18:00). The cage floor was covered with aspen wood bedding (Tapvei, Estonia). Commercial pelleted rat chow (RM1 (E) SQC Expanded, SDS Diets, Witham, Essex, England) and filtered, UV-irradiated tap water were available ad libitum. The animal room was air conditioned with the temperature being kept at 22 ± 1 °C, and relative humidity at 38–75% (typically 50%).

Rats were divided into two separate experiments. In both, rats were matched for body weight and randomly allocated to one of two treatment groups (Figure 1, Table S1). In the first experiment (acute exposure), rats (*n* = 6 per group; housed singly) were treated with either a single dose of C2 (100 mg/kg dissolved in PEG-400, the highest possible dose as previously determined [42], at a total volume of 5 mL/kg to minimize adverse effects [42]) or vehicle control alone (PEG-400; 5 mL/kg) by oral gavage. The rats were euthanized by carbon dioxide 1 day following exposure. In the second (subacute) experiment, rats (*n* = 6 per group; housed in groups of 2–3) were administered repeated doses of C2 (100 mg/kg/day, as above) or vehicle control (PEG-400, 5 mL/kg/day) daily for 5 consecutive days (days 1–5) and euthanized on day 10. A single animal from the C2-treated group was lost due to accidental death during the exposure phase. As the half-life of C2 is ~1.7 h, these dosing regimens allowed for identification of transcriptomic changes (secondary and indirect effects) following brief (acute) or repeated (subacute) exposures. Furthermore, these time points (1 day and 10 day exposures) enabled comparison with previous studies on the transcriptomic impact of TCDD exposure for 1 or 10 days in rat liver [45,59]. Liver tissue was extracted and frozen in liquid nitrogen. It was then stored at −80 °C until analysis.

### 4.2. Data Generation and Preprocessing

Total RNA was isolated using the Sigma-Aldrich GenElute™ Mammalian total RNA isolation kit at the University of Helsinki (Finland). RNA aliquots were then sent to The Centre for Applied Genomics (TCAG) at The Hospital for Sick Children (Toronto, Canada). RNA quality was verified by electrophoresis using RNA 6000 Nano kits on an Agilent 2100 Bioanalyzer and assayed on Affymetrix GeneChip Rat Gene 2.0 ST arrays using the manufacturer’s protocols.

Raw microarray data (CEL files) were analyzed using the Affy package (v1.48.0) in the R statistical environment (v3.4.3), using the RMA algorithm [76]. Probes were mapped to Entrez Gene IDs using the custom cdf ragene20strnentrezgcdf (v21.0.0) package for R [77]. Distributional and spatial homogeneity of arrays was assessed (Figure S2); no outliers were detected visually.

### 4.3. Statistical Analysis and Visualizations

A standard linear model was performed, with contrasts fit to identify genes with altered RNA abundance between C2-exposed and control rat liver for each treatment group. The standard error of each coefficient was adjusted with an empirical Bayes moderation of standard error [78]. Model-based *t*-tests were used to test for difference in significance to zero, which was followed by a false-discovery rate (FDR) adjustment for multiple hypothesis testing [79]. Modeling was performed using the limma (v3.32.10) package for R. A *p*-value sensitivity analysis was used to determine an appropriate significance threshold used for downstream analyses (FDR < 0.01). All visualizations were generated using the BPG package for R (v5.9.4) [80], leveraging the lattice (v0.20-35) and latticeExtra (v0.6-28) packages, and with the VennDiagram package [81] used to visualize overlap.

### 4.4. Transcription Factor Binding Site (TFBS) Analysis

A transcription factor binding site analysis was performed to target motifs associated with AHR transcriptional regulation. The rat reference genome (rn6) was searched for given motif sequences occurring within ±3 kbp of the transcription start site of each gene. RefLink and RefFlat tables were downloaded from the UCSC genome browser to annotate transcription start sites (2018-08-09). Four motifs were examined—AHRE-1 (core), AHRE-1 (extended), AHRE-1 (full), and AHRE-2—with sequences GCGTG, TNGCGTG, [T|G]NGCGTG[A|C][G|C]A, and CATG{N6}C[T|A]TG, respectively [82,83].

### 4.5. Pathway Analysis

Genes with statistically different mRNA abundance changes in C2-exposed rat liver relative to control (FDR < 0.05, |fold change| > 0.5) were selected for pathway analyses and submitted to the High-Throughput GoMiner web interface (application build 469, database build 2011-01) [84]. Each gene list was compared against a randomly drawn sample from all other genes in the dataset, using an FDR threshold of 0.1, 1000 randomizations, all rat databases and look-up options, and all GO evidence codes and ontologies (Table S2). Significantly enriched pathways were further selected using a threshold of *p_adj_* < 0.01 and an enrichment score > 5 in at least one of the experimental groups. This resulted in 19 unique gene ontologies to examine further.

### 4.6. Comparison to TCDD

The TCDD.Transcriptomics (v2.1.1) [22] package for R provided fully processed data from numerous microarray studies of TCDD-exposed rat and mouse tissues. Data generated from hepatic tissue of TCDD-sensitive Long–Evans (*Turku/AB*; L-E) and TCDD-resistant Han/Wistar (*Kuopio*; H/W) rats following a single exposure to TCDD and collected 19 h or 10 days later were used for comparison to the acute and subacute C2 exposure groups, respectively.

### 4.7. Data Availability

All data have been deposited in the Gene Expression Omnibus (GSE126216) and are publically available. Raw, normalized, and modeled data are also available in the TCDD Transcriptomics package for R (v2.2.5; available for download from http://labs.oicr.on.ca/boutros-lab/tcdd-transcriptomics) [22].

## Figures and Tables

**Table 1:** Differential transcriptomic response of carbonic anhydrases by C2 and 2,3,7,8-tetrachlorodibenzo-*p*-dioxin (TCDD).

	Treatment	Sprague-Dawley (C2)	Time Point	H/W (TCDD)	L-E (TCDD)
*Car1*	Acute	−0.25	19 h	−0.29 *	−0.19
	Subacute	−1.18 ***	4 days	−0.87 ***	−0.05
			10 days	−2.24 ***	−0.16
*Car3*	Acute	−0.22	19 h	−0.64	−1.38 **
	Subacute	−0.48 **	4 days	0.04	−5.08 ***
			10 days	−0.68	−5.13 ***

Two carbonic anhydrase genes (*Car1* and *Car3*) demonstrated largely different response patterns in rat liver following exposure to either C2 (Sprague-Dawley rats; acute or subacute exposure) or TCDD (Han/Wistar and Long–Evans rats; 19 h, 4, or 10 days after exposure). Values indicate log_2_ fold changes; * indicates FDR-adjusted *p*-value < 0.05; ** FDR < 0.01; *** FDR < 0.001.

## Supplementary Materials

Supplementary materials can be found at https://www.mdpi.com/1422-0067/20/6/1370/s1.

## Abbreviations

AHR	Aryl hydrocarbon receptor
SAHRM	selective AHR modulator
LAQ	Laquinimod
DELAQ	Deethylated laquinimod
TCDD	2,3,7,8-tetrachlorodibenzo-*p*-dioxin
C2	IMA-08401; N-acetyl-N-phenyl-4-acetoxy-5-chloro-1,2-dihydro-1-methyl-2-oxo-quinoline-3-carboxamide
AHRE	AHR response elements
H/W	Han/Wistar (*Kuopio*)
L-E	Long–Evans (*Turku/AB*)
