# Supplementary material for: Transcriptomic Impact of IMA-08401, a Novel AHR Agonist Resembling Laquinimod, on Rat Liver
Source: Int J Mol Sci. 2019 Mar 19;20(6):1370. doi: 10.3390/ijms20061370 (PMC6471016; doi:10.3390/ijms20061370)
Supplement: Supplementary file 1 [file ijms-20-01370-s001.zip › ijms-460511 supplementary.docx]

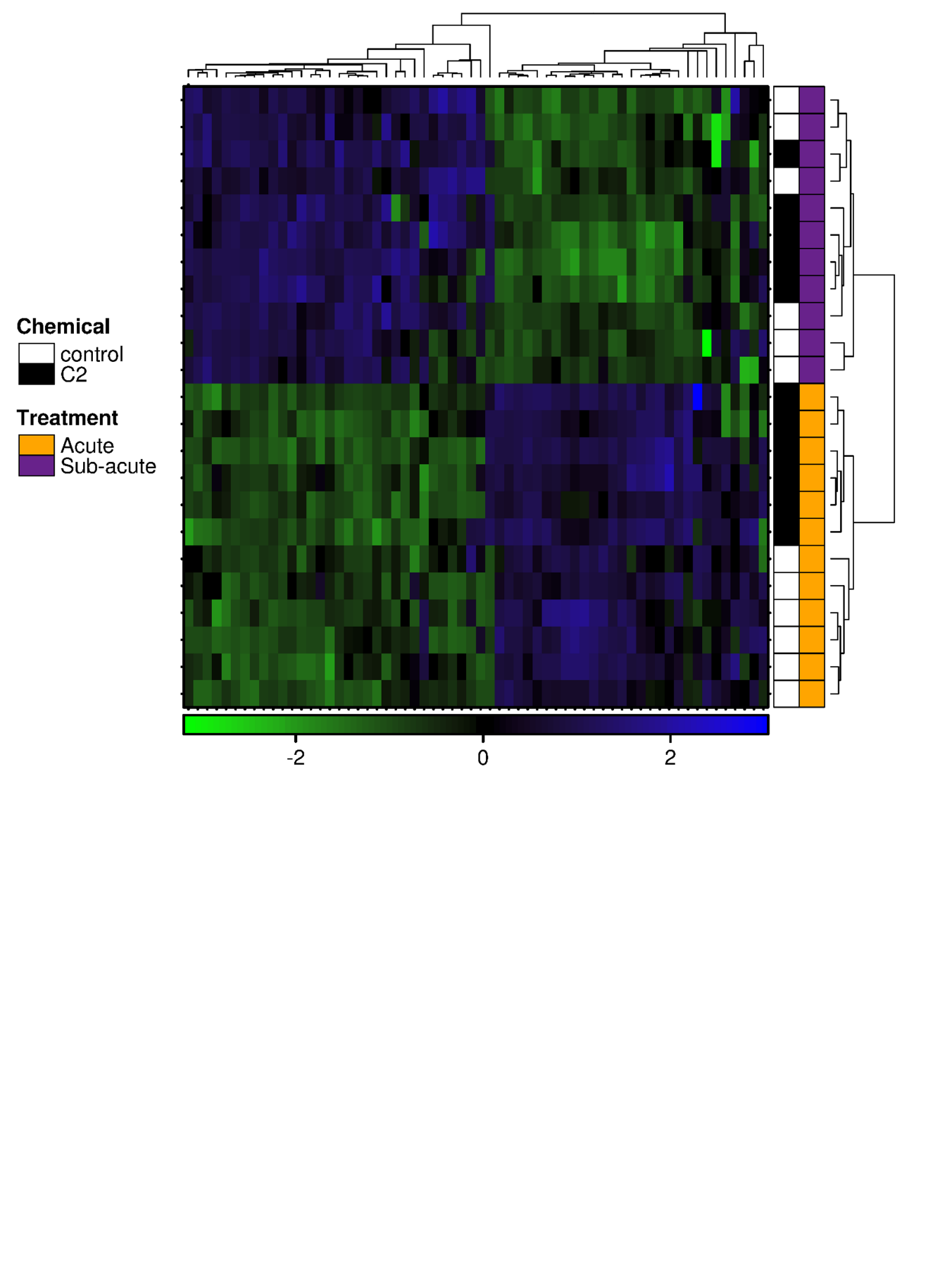


**Figure S1.** Transcriptomic profiles of C2 exposed rat liver. Variance among RMA normalized intensity values was assessed and top variably expressed genes were identified as those with variance > 1 across the cohort.


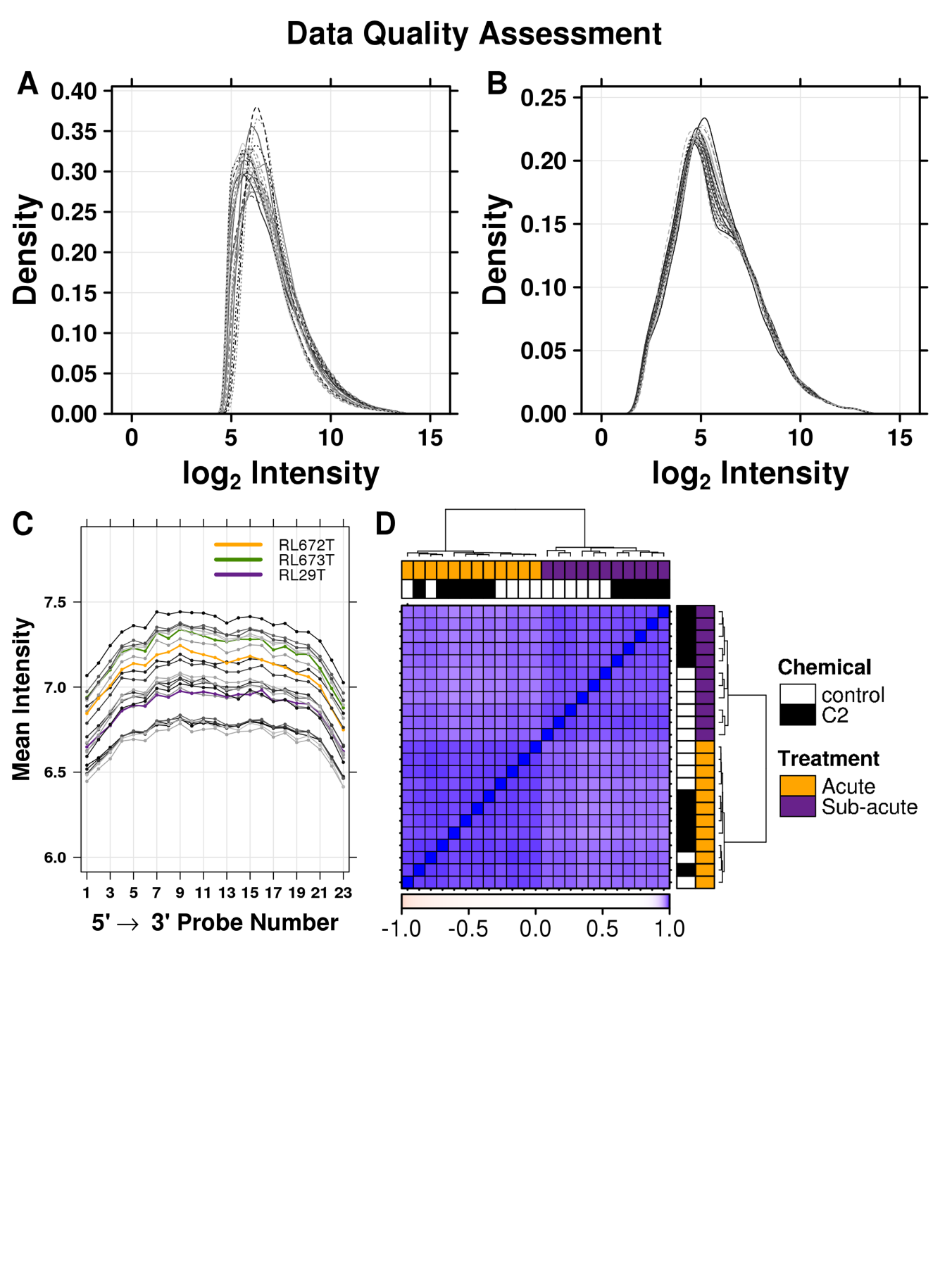


**Figure S2.** Quality Assessment of Microarrays. All arrays were normalized simultaneously; distribution of intensity values A) pre- and B) post- RMA normalization. C) RNA degradation was evaluated by examination of intensity estimates across each probe. D) Heatmap demonstrating inter-array correlation; clustering was performed using the DIANA algorithm with a Pearson’s correlation similarity metric; no outliers were detected by any of these metrics.
